# Prognostic indices for brain metastases – usefulness and challenges

**DOI:** 10.1186/1748-717X-4-10

**Published:** 2009-03-04

**Authors:** Carsten Nieder, Minesh P Mehta

**Affiliations:** 1 Medical Department, Division of Oncology, Nordland Hospital, 8092 Bodø, Norway; 2 Faculty of Medicine, Institute of Clinical Medicine, University of Tromsø, 9038 Tromsø, Norway; 3 Department of Human Oncology, University of Wisconsin Hospital Medical School, Madison, WI 53792, USA

## Abstract

**Background:**

This review addresses the strengths and weaknesses of 6 different prognostic indices, published since the Radiation Therapy Oncology Group (RTOG) developed and validated the widely used 3-tiered prognostic index known as recursive partitioning analysis (RPA) classes, i.e. between 1997 and 2008. In addition, other analyses of prognostic factors in groups of patients, which typically are underrepresented in large trials or databases, published in the same time period are reviewed.

**Methods:**

Based on a systematic literature search, studies with more than 20 patients were included. The methods and results of prognostic factor analyses were extracted and compared. The authors discuss why current data suggest a need for a more refined index than RPA.

**Results:**

So far, none of the indices has been derived from analyses of all potential prognostic factors. The 3 most recently published indices, including the RTOG's graded prognostic assessment (GPA), all expanded from the primary 3-tiered RPA system to a 4-tiered system. The authors' own data confirm the results of the RTOG GPA analysis and support further evaluation of this tool.

**Conclusion:**

This review provides a basis for further refinement of the current prognostic indices by identifying open questions regarding, e.g., performance of the ideal index, evaluation of new candidate parameters, and separate analyses for different cancer types. Unusual primary tumors and their potential differences in biology or unique treatment approaches are not well represented in large pooled analyses.

## Background

Prognostic indices might represent a useful tool in palliative cancer treatment. Estimation of a patient's prognosis in terms of overall survival might allow for tailored treatment, i.e. more aggressive approaches when these are likely to impact on survival and focus on disease stabilisation, symptom control and toxicity minimization when the disease is more advanced, or comorbidity limits the tolerability of aggressive therapy. In addition, prognostic indices might also be used as inclusion/exclusion criteria for clinical trials and for comparison of results across different studies in relatively homogeneous patient groups.

Brain metastases continue to represent a formidable challenge in oncology [1-3]. With increasing numbers of local and systemic treatment options, the issue of patient selection gains importance. While surgery and stereotactic radiosurgery (SRS) provide long-term local control of macroscopic disease and in combination with whole-brain radiotherapy (WBRT) the best available overall brain control for the remaining life time [4-10], they represent overtreatment in patients with short survival, which typically is caused by uncontrollable systemic disease. This review will address the strengths and weaknesses of 6 different prognostic indices, published since the Radiation Therapy Oncology Group (RTOG) developed and validated the widely used 3-tiered prognostic index known as recursive partitioning analysis (RPA) classes [11,12], i.e. between 1997 and 2008. In addition, other analyses of prognostic factors in groups of patients, which typically are underrepresented in large trials or databases, published in the same time period are reviewed. These include patients with primary tumors that do not commonly metastasize to the brain, and the elderly, who are often either excluded or under-represented in clinical trials.

## Methods

The present review compares different prognostic indices and analyses of prognostic factors based on a systematic literature search by use of Medline (Pub Med by the National Library of Medicine, National Institutes of Health, Bethesda, Maryland, USA). It is limited to adult patients having received first-line treatment for parenchymal brain metastases in the absence of leptomeningeal disease. The key words used were "brain metastases", "metastatic brain tumor" and "cerebral metastases". The final search was performed on June 30, 2008. It also included the reference lists of all articles and the appropriate chapters in textbooks on brain metastases, neuro-oncology and radiation oncology. Case reports and review articles were not assessed. Only studies with more than 20 patients were included. If several subsequent reports were published from the same institution, the most recent publication was evaluated. The methods and results of prognostic factor analyses were extracted and compared.

## Results

The search identified 6 different prognostic indices, which are shown in Table 1. Comparison of the patients' characteristics is shown in Table 2. Unfortunately, a considerable amount of information can not be extracted from the publications. The most widely used index over the last decade is the RPA index originally described by Gaspar et al. on behalf of the RTOG [11], which is based on 4 parameters (age, Karnofsky performance status (KPS), presence or absence of extracranial metastases, and the control status of the primary tumor), separating patients into 3 different classes. Lutterbach et al. suggested expansion of the classification by further dividing class III into 3 separate classes [13]. This was based on their multivariate analysis of 916 patients from a single institution, but was not adopted by other authors in subsequent publications. Their definition yielded class IIIa defined as age <65 years, controlled primary tumor and single brain metastasis, class IIIc defined as age ≥ 65 years, uncontrolled primary tumor and multiple brain metastases, while other patients would make up class IIIb. The original RPA classification has been validated by several authors, both in selected and unselected patient groups, e.g., patients with breast primary, lung primary (small cell and non-small cell), malignant melanoma, unknown primary, or surgical resection and SRS as main local treatment modalities [14-35].

**Table 1:** Comparison of the prognostic scores published since 1997, empty fields indicate that a parameter is not used in the index

Score	Performance status	Age	Extracranial metastases	Controlled primary	Steroid treatment	Number of BM	Volume of BM	Interval to BM	Class I	Class II	Class III	Class IV
RPA^11^ Derived from 3 prospective RTOG studies, n = 1,200	KPS ≥ 70 vs <70	<65 years	no vs yes	no vs yes					all 4 favourable factors	other patients	KPS <70	none
Rotterdam^36^ Single institution, n = 1,292	ECOG 0–1 vs 2–3		limited activity vs	systemic extensive*	good, moderate or little response				ECOG 0–1 with no or limited systemic tumor activity and good response to steroids	other patients	ECOG2-3 with limited or extensive systemic activity and little response to steroids	none
SIR^37^ Single institution, n = 65	KPS 80–100:2 points KPS 60–70: 1 point KPS ≤ 50: 0 points	≤ 50: 2 points 51–59: 1 point ≥ 60: 0 points	no evidence of systemic disease or complete remission: 2 points partial remission or stable	disease: 1 point progressive disease: 0 points		1: 2 points 2: 1 point ≥ 3: 0 points	largest lesion volume <5 cc: 2 points 5–13 cc: 1 point >13 cc: 0 points		8–10 points	4–7 points	1–3 points	none
BSBM^43^ Single institution, n = 110	KPS 80–100: 1 point KPS ≤ 70: 0 point		no: 1 point yes: 0 points	yes: 1 point no: 0 points					3 points	2 points	1 point	0 points
GPA^44^ Derived from 5 prospective RTOG studies, n = 1,960	KPS 90–100: 1 point KPS 70–80: 0.5 points KPS <70: 0 points	<50: 1 point 50–59: 0.5 points >60: 0 points	none: 1 point present: 0 points			1: 1 point 2–3: 0.5 points >3: 0 points			3.5–4 points	3 points	1.5–2.5 points	0–1 points
Rades et al.^45^ Multi-institutional, n = 1,085	KPS ≥ 70: 5 points KPS <70: 1 point	≤ 60: 4 points >60: 3 points	none: 5 points present: 2 points					>8 mo: 4 points ≤ 8 mo: 3 points	17–18 points	14–16 points	11–13 points	9–10 points

BM: brain metastases, RPA: recursive partitioning analysis, RTOG: Radiation Therapy Oncology Group, KPS: Karnofsky performance score, SIR: score index for radiosurgery, BSBM: basic score for brain metastases, GPA: graded prognostic assessment, ECOG: Eastern Cooperative Oncology Group

* limited systemic activity: no systemic metastases but progression of primary tumor or systemic metastases with primary tumor absent or controlled; extensive systemic activity: systemic metastases and progressive primary

**Table 2:** Median values of reported patients' characteristics in each of the studies, empty fields indicate missing information

Score	Performance status	Age	Extracranial metastases	Controlled primary	Steroid treatment	Number of BM	Volume of BM	Interval to BM
RPA^11^ n = 1,200	KPS 70	55–59 yrs. range	38%	60%		2		
Rotterdam^36^ n = 1,292	ECOG 1	59 yrs.			mean 15 mg dexamethasone per day	2		8.5 mo.
SIR^37^ n = 65	KPS 80	61 yrs.				2	3.3 cc	
BSBM^43^ n = 110		57 yrs.				2	9 cc	
GPA^44^ n = 1,960	KPS 80	60 yrs.	36%	67%		2	5–13 cc	
Rades et al.^45^ n = 1,085	KPS 70	60 yrs.	64%					8 mo.

BM: brain metastases, RPA: recursive partitioning analysis, KPS: Karnofsky performance score, SIR: score index for radiosurgery, BSBM: basic score for brain metastases, GPA: graded prognostic assessment, ECOG: Eastern Cooperative Oncology Group

**Figure 1:**
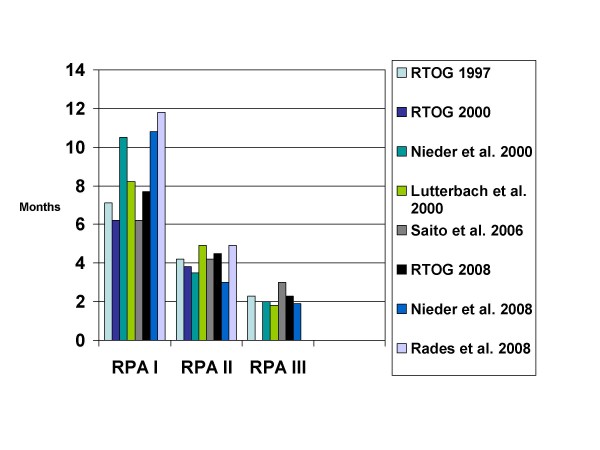
**Comparison of median survival in 7 studies using the recursive partitioning analyses (RPA) classes (treatment was WBRT with or without local measures, none of the studies is limited to one particular cancer type)**.

Probably, the surgically treated patients represent the most homogeneous cohorts assessed with the RPA system, as these were patients with rather favourable prognosis, fit to undergo surgery and with limited brain disease. Nevertheless, the differences in median survival between the individual studies were large. In RPA class I, median survival ranged from 15–29 months [31-35]. In class II, a survival range of 5.5–11 months has been reported. In class III, these figures reached 1.4–9 months. As illustrated here, survival within the same RPA class might vary by a factor of 2 or more between different studies (identical treatment approach). In series where the majority of patients were treated with WBRT, less variation between studies can be found (Figure 1). As shown in Table 1, both RPA class II and III contain quite heterogeneous groups of patients. The factor determining class III is KPS<70, which might result from many different causes including the brain metastases themselves, advanced and treatment-refractory extracranial metastases, severe pain or pathological fracture in patients with bone metastases, atelectasis or pneumonia from primary lung cancer, anemia induced by chemotherapy, recovery from recent surgery, and non-cancer-related comorbidity. In all the reports reviewed variable proportions of patients in the most favourable RPA class I unexpectedly died within 2 months, while some patients in class III survived for more than 6 months. For these reasons, there obviously is a need for a more refined index than RPA.

**Figure 2:**
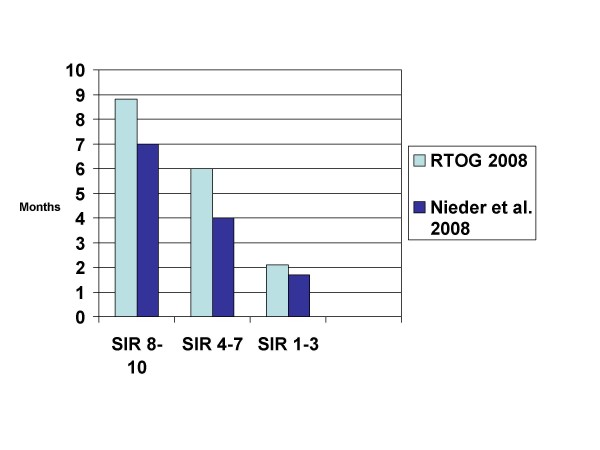
**Comparison of median survival in 2 studies using the score index for radiosurgery (SIR) (treatment was WBRT with or without local measures, studies not limited to one particular cancer type)**.

The first attempt in 1999 resulted in the Rotterdam Score, which did not gain wider acceptance [36]. Similar to RPA, performance status and extent of systemic disease were included, while the third parameter was response to steroids before WBRT. It can be assumed that the unavailability of this latter parameter in most databases or patient records prevented other groups from using the score. In addition, the definition of systemic tumor activity is not straight forward. The next attempt (Score Index for Radiosurgery (SIR)) was derived from a limited number of patients treated with this particular focal approach, which might have resulted in overfitting of the data [37]. However, several groups confirmed the performance of the SIR in patients treated with SRS, surgery, and WBRT with or without SRS, some of them with large numbers of patients (Figure 2) [35,38-44]. To accurately define systemic disease activity, comprehensive diagnostic work-up is needed.

**Figure 3:**
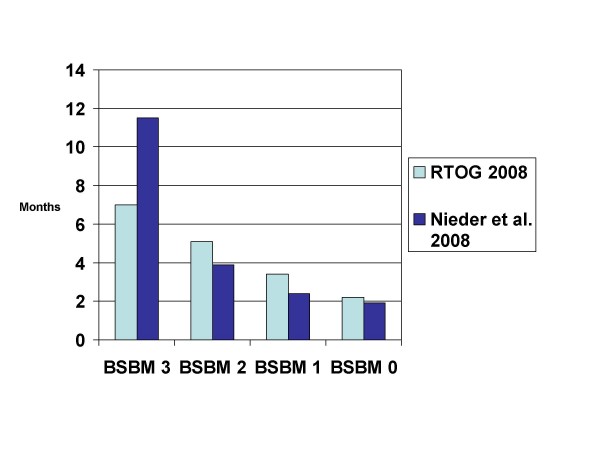
**Comparison of median survival in 2 studies using the basic score for brain metastases (BSBM) (treatment was WBRT with or without local measures, studies not limited to one particular cancer type)**.

When evaluating the SIR and RPA indices in their SRS database, the group from Brussels, Belgium, arrived at a new score, which they called Basic Score for Brain Metastases (BSBM) [43]. Based on its greater convenience and simplicity, they advocated the use of this score, which uses the same definition of extracranial disease activity as the RTOG. Recent data indicate that BSBM can be applied to patients managed with WBRT with or without SRS and surgery plus WBRT [35,42,44], however its performance is not better than that of the other scores (Figure 3).

**Figure 4:**
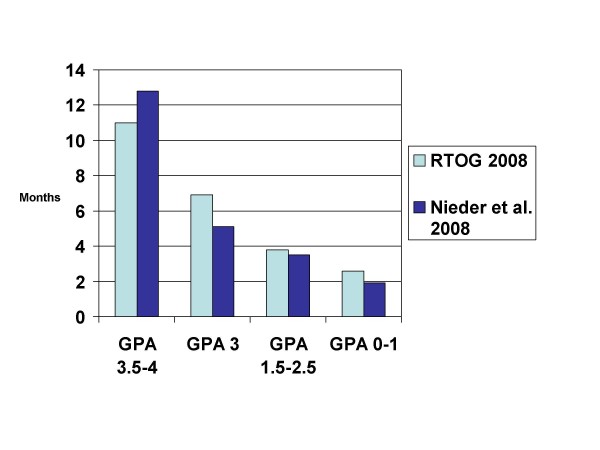
**Comparison of median survival in 2 studies using the graded prognostic assessment (GPA) (treatment was WBRT with or without local measures, studies not limited to one particular cancer type)**.

The RTOG has recently proposed a new index, which was compared to RPA, SIR, and BSBM (but not to the Rotterdam score) [44]. The new score (Graded Prognostic Assessment (GPA)) is different from RTOG's RPA, e.g., with regard to the number of prognostic classes, which increased from 3 to 4, and the larger number of patients. The analysis also includes patients managed with WBRT plus SRS from RTOG study 9508 [5]. In the GPA system, 3 different values (0, 0.5 or 1) are assigned for each of these 4 parameters: age (≥ 60; 50–59; <50), KPS (<70; 70–80; 90–100), number of brain metastases (>3; 2–3; 1), and extracranial metastases (present; not applicable; none). Assessment of primary tumor activity or control is no longer mandated. It was concluded by the authors that "GPA is the least subjective, most quantitative and easiest to use of the 4 indices" and that future trials should compare these scores and validate the GPA. One of the authors' group has embarked on this comparison in 2 different patient populations, i.e. those managed with WBRT with or without SRS (comparable to the RTOG study population) [42] and those managed with surgery and WBRT [35]. Both studies basically relied on the methods used by the RTOG in their analysis, though with patients treated in clinical routine outside of randomized trials. Compared to RTOG's patients treated with WBRT with or without SRS, the median age, KPS, number of lesions and lesion volume were similar. Obvious differences existed, however, regarding controlled primary tumor (47 vs. 67%) and extracranial metastases (56 vs. 36%). Thus, the cohort is expected to have inferior survival. Figure 4 shows the survival results.

**Figure 5:**
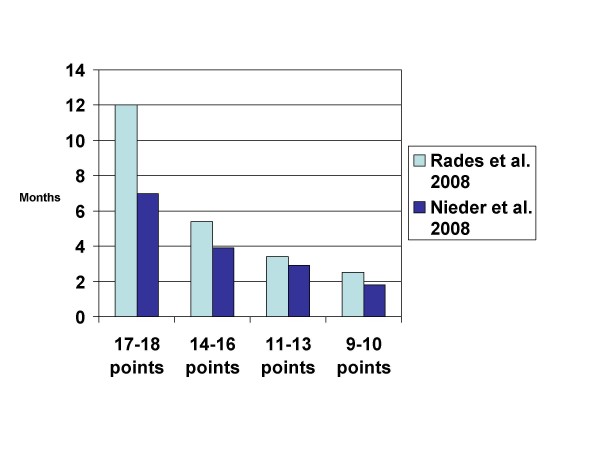
**Comparison of median survival in 2 studies using the index proposed by Rades et al. **[45]**(treatment was WBRT with or without local measures, studies not limited to one particular cancer type, median survival estimated from the Kaplan-Meier curves in the publication)**.

Last but not least, Rades et al. developed a new prognostic index based on 4 parameters (age, KPS, extracranial metastases at the time of WBRT, interval from tumor diagnosis to WBRT) [45]. The major difference from the RPA classes is the replacement of primary tumor control by interval from tumor diagnosis to WBRT (not by number of brain metastases as in the GPA). This index separated patients into 4 subgroups with significantly different prognosis and was also validated in one of the authors' database (unpublished results, Figure 5).

## Discussion

As stated on the website of the National Cancer Institute , a prognostic factor is regarded as a situation or condition, or a characteristic of a patient, that can be used to estimate the chance of recovery from a disease or the chance of the disease recurring. Based on such prognostic factors, 6 different prognostic indices for adult patients with brain metastases from solid tumors have been developed over the last decade. As demonstrated in Table 1, the 3 most recently published indices all expanded from the primary 3-tiered RPA system to a 4-tiered system. The 6 indices are based on a different number of prognostic factors, i.e. 3–6. Of course, increasing numbers of parameters will lead to less convenience and ease of administration. None of the groups that developed these indices included all potential prognostic factors in their analysis. This is most likely due to the unavailability of all the information in the databases and the difficulty in collecting missing data in 1,000 or more patients treated over many years. As can be seen in Figures 3, 4, 5, the performance of the 4-tiered indices is not tremendously different, although further data are needed to confirm this finding.

There is agreement in all indices on the importance of performance status and extracranial disease activity. However, whether both primary tumor and extracranial metastases should be considered is less clear (2 indices would not include primary tumor control). Assessment of extracranial disease status is not trivial. It might require considerable resources in patients with very limited life expectancy and therapeutic options. When collecting data over long time periods, one must expect a shift in diagnostic modalities, i.e. increasing use of magnetic resonance imaging of the brain as compared to computed tomography (CT) or increasing use of chest CT or even positron emission tomography (PET). Such a shift will likely result in no longer assigning patients to the most favourable prognostic class (stage migration). This might compromise the comparison of the different studies.

Two of the 6 indices did not include age and the ones that did, used slightly different cut-off values. A minority of studies (n = 2) included number of brain metastases and only one each included response to steroids, volume of the largest lesion in the brain, and time interval to development of brain metastases, respectively. Other previous reports lend credence to the examination of each of these factors. In their multivariate analysis of 334 patients, DiLuna et al. reported significantly better survival in patients with 1–3 vs 4 or more brain metastases and in those patients with both limited number and volume of brain metastases (<5 cc total volume) [46]. Bhatnagar et al. also reported on the impact of treatment volume as independent prognostic factor in patients treated with SRS [47]. In a randomised trial with 544 patients, Priestman et al. found that dose of steroids was independently associated with survival [48]. Interval to development of brain metastases appears particularly important in patients with primary NSCLC and malignant melanoma. The multivariate analyses of 3 studies with 292–686 patients support this observation [23,49,50].

The latter findings lead to the general question on the usefulness of lumping together patients with different primary tumors in these models. Breast cancer poses an interesting dilemma here, because although tumor type and histology were not prognostically significant in the RPA, recent data, especially since the advent of trastuzumab and lapatinib, suggest that, receptor status and her-2-neu expression might have prognostic impact, even if this issue is not without controversy (Table 3). The recently suggested prognostic factor lymphopenia falls into the same category [19,51]. Unusual primary tumors and their potential differences in biology or unique treatment approaches are not well represented in large pooled analyses. Table 4 provides examples on analyses of prognostic factors in such groups.

**Table 3:** Prognostic impact of hormone receptor and HER-2 status in patients with brain metastases from breast cancer

	*n*	Prognostic impact of hormone receptor status	Prognostic impact of HER-2 status
Claude et al.^51^	120	none	not examined
Bartsch et al.^52^	174	none	None
Le Scodan et al.^19^	117	receptor negative significantly worse	None
Nam et al.^53^	126	receptor negative significantly worse	HER-2 negative significantly worse
Kirsch et al.^54^	95	not examined	HER-2 negative significantly worse*
Eichler et al.^55^	83	none	HER-2 negative significantly worse^^^
Melisko et al.^56^	112	receptor negative significantly worse	none
Harputluoglu et al.^57^	144	none	none
Park et al.^58^	125	none	HER-2 positive significantly worse
Church et al.^59^	86	not examined	HER-2 negative significantly worse*

^^^80% of HER-2 overexpressing cases received trastuzumab after diagnosis of brain metastases

* the difference in survival was limited to patients with HER-2 overexpressing cancer treated with trastuzumab after diagnosis of brain metastases

**Table 4:** Prognostic factors in patients underrepresented in large studies (minimum number of patients n = 20)

Author	Population	Significant prognostic factors
Ogawa et al.^60^	esophageal cancer, n = 36	KPS, aggressive local treatment (multivariate)
Weinberg et al.^61^	esophageal cancer, n = 27	no liver metastases, RPA class I (trend, p = 0.1, multivariate)
Khuntia et al.^62^	esophageal cancer, n = 27	KPS, aggressive local treatment (multivariate)
Cohen et al.^63^	ovarian cancer, n = 72	aggressive local treatment
Cormio et al.^64^	ovarian cancer, n = 22	extracranial disease, time to development of brain metastases
Growdon et al.^65^	gynaecological cancers, n = 30	extracranial disease, histology, use of chemotherapy (multivariate)
Tremont-Lukats et al.^66^	prostate cancer, n = 103	adenocarcinoma vs other histology
Rades et al.^24^	unknown primary, n = 101	KPS, extracranial metastases, RPA class
Bartelt and Lutterbach^67^	unknown primary, n = 47	KPS, surgical resection status (multivariate)
Ruda et al.^68^	unknown primary, n = 33	number of brain metastases (multivariate)
Kim et al.^69^	patients ≥ 75 years, SRS treatment, n = 44	single brain metastasis, NSCLC vs other primary
Noel et al.^70^	patients ≥ 65 years, SRS treatment, n = 117	KPS (multivariate)

WBRT: whole-brain radiotherapy, KPS: Karnofsky performance status, RPA: recursive partitioning analysis, SRS: stereotactic radiosurgery, NSCLC: non-small cell lung cancer

Surrogate markers of disease activity that are easy to measure and inexpensive, such as lactate dehydrogenase and other laboratory parameters have repeatedly been shown to be independent prognostic factors for survival [71-74]. Studies that were not limited to patients with brain metastases suggest that the anorexia-cachexia syndrome, dyspnea, pain, and co-morbidity are further candidates for prospective evaluation [74]. The same holds true for neurofunction class [75,76] and mini mental status examination results, which was an independent prognostic factor for survival in a multivariate model that also included KPS [77]. The current prognostic indices unfortunately do not incorporate these features.

One of the purposes of prognostic indices is to guide the choice of treatment in individual patients. In this context, a prognostic index should be accurate enough to avoid overtreatment in patients that actually have very short survival. Even more important, one should not withhold treatment because the index erroneously predicts an unfavorable outcome. These aspects of the indices have not been thoroughly evaluated, even in the recent GPA analysis [44]. In our analysis of 239 patients, which confirms that RPA, SIR, BSBM and GPA each split the dataset into groups with significantly different prognosis, this issue was addressed [42]. With regard to the outcome of patients with unfavorable survival, defined as ≤ 2 months (n = 93), no significant difference between the indices was observed. Regarding patients with favorable survival, defined as ≥ 6 months (n = 66), again no significant difference was observed, although RPA performed worse than the other indices. Overall, GPA misassigned 6% of the patients (9 out of 159), compared to 11% with RPA. Therefore, the available validation data certainly do not discourage further evaluation of the new GPA. However, such evaluation should also include comparison with the 2 other scores (Rotterdam and Rades et al.). It is just the stark reality of the disease process that in all of the scoring systems, the most favorable prognostic group is very small (e.g., GPA ≥ 3.5: 9% of RTOG and 7% of our own patients; RPA class I: 16% of RTOG and 11% of our own patients).

The open questions after publication of 6 prognostic indices include:

- how should the ideal index perform?

- how many parameters should form the basis of the ideal index?

- can we lump together patients with breast cancer, small-cell lung cancer, malignant melanoma etc. or do we lose potentially important information?

- do we need candidate parameters beyond the ones examined so far (lactate dehydrogenase, anemia, weight loss, pain etc.)?

- is it justifiable to assign the same point value to different degrees of extracranial disease, e.g., 2 small asymptomatic lung metastases, 8 large liver metastases with increased bilirubin, skin metastases already treated by radiotherapy etc.?

- can international groups collaborate to develop a consensus score, or maybe even an online tool?

Other aspects of predicting the outcome in patients with brain metastases that many clinicians might appreciate, relate to the important issue of neurologic function and quality of life. In many instances, radiotherapy aims more on improving deficits and preventing neurologic decline than prolonging survival, but no attempts have been made to develop scores that address endpoints other than overall survival. It appears therefore worthwhile to collect data on such endpoints, as done, e.g., in the recently completed randomized trial of radiotherapy with or without motexafin gadolinium [78], which used time to neurologic progression as primary endpoint. Other opportunities for future research include examination of prognostic models that provide estimates on both risk of systemic cancer progression with death from non-neurologic causes and risk of death from uncontrolled brain metastases.

## Competing interests

The authors declare that they have no competing interests.

## Authors' contributions

CN and MM drafted the manuscript and participated in the design of the study. Both authors read and approved the final manuscript.
